# Triterpenoid herbal saponins enhance beneficial bacteria, decrease sulfate-reducing bacteria, modulate inflammatory intestinal microenvironment and exert cancer preventive effects in *Apc^Min/+^* mice

**DOI:** 10.18632/oncotarget.8886

**Published:** 2016-04-21

**Authors:** Lei Chen, Manreetpal S. Brar, Frederick C. C. Leung, W. L. Wendy Hsiao

**Affiliations:** ^1^ Center for Cancer & Inflammation Research, School of Chinese Medicine, Hong Kong Baptist University, Kowloon, Hong Kong, China; ^2^ School of Biological Sciences, University of Hong Kong, Pokfulam, Hong Kong, China; ^3^ State Key Laboratory of Quality Research in Chinese Medicine, Macau University of Science and Technology, Taipa, Macau, China

**Keywords:** herbal saponins, gynostemma pentaphyllum, pyrosequencing, gut microbiota, gut mucosal environment

## Abstract

Saponins derived from medicinal plants have raised considerable interest for their preventive roles in various diseases. Here, we investigated the impacts of triterpenoid saponins isolated from *Gynostemma pentaphyllum* (GpS) on gut microbiome, mucosal environment, and the preventive effect on tumor growth. Six-week old *Apc^Min/+^* mice and their wild-type littermates were fed either with vehicle or GpS daily for the duration of 8 weeks. The fecal microbiome was analyzed by enterobacterial repetitive intergenic consensus (ERIC)-PCR and 16S rRNA gene pyrosequencing. Study showed that GpS treatment significantly reduced the number of intestinal polyps in a preventive mode. More importantly, GpS feeding strikingly reduced the sulfate-reducing bacteria lineage, which are known to produce hydrogen sulfide and contribute to damage the intestinal epithelium or even promote cancer progression. Meanwhile, GpS also boosted the beneficial microbes. In the gut barrier of the *Apc^Min/+^* mice, GpS treatment increased Paneth and goblet cells, up-regulated E-cadherin and down-regulated N-cadherin. In addition, GpS decreased the pro-oncogenic β-catenin, p-Src and the p-STAT3. Furthermore, GpS might also improve the inflamed gut epithelium of the *Apc^Min/+^* mice by upregulating the anti-inflammatory cytokine IL-4, while downregulating pro-inflammatory cytokines TNF-β, IL-1β and IL-18. Intriguingly, GpS markedly stimulated M2 and suppressed M1 macrophage markers, indicating that GpS altered mucosal cytokine profile in favor of the M1 to M2 macrophages switching, facilitating intestinal tissue repair. In conclusion, GpS might reverse the host's inflammatory phenotype by increasing beneficial bacteria, decreasing sulfate-reducing bacteria, and alleviating intestinal inflammatory gut environment, which might contribute to its cancer preventive effects.

## INTRODUCTION

Human gut harbors 100 trillion microbial organisms that is intrinsically linked to individual's health and diseases. In the gut of mammalian hosts, microbiota is found to engage a dynamic interaction with the host immune cells residing at the surface of the intestinal tract where the commensal bacteria colonize. The host-microbes are maintained in a symbiotic stage. Such interaction may lead to an optimal inflammatory cytokine release by the host immune cells in order to regulate the resident microbiota, and block the foreign pathological bacteria. Such delicate and dynamic homeostasis can be deregulated by various factors in the gut microenvironment and results in prolonged inflammation in the gut. This persistent inflammation may account for the link between the gut microbiota and various chronic inflammatory diseases, including colonic carcinogenesis.

Pyrosequencing of 16S rRNA gene on gut microbiome has revealed a clear structural segregation of gut microbiota between colorectal cancer (CRC) patients and healthy volunteers [1–3]. The impact of commensal microbiota on CRC development has also been investigated in several animal models by using germ-free mice and microbiota transplantation experiments [4–7]. Several review articles have well documented how gut microbes cross with diet, inflammation, and host genetics to influence CRC development [8–10]. Tjalsma *et al.* [11] hypothesized that CRC is initiated by particular bacterial drivers and promoted by certain bacterial passengers. Indeed, few of the commensal microbiota has been highlighted in recent studies for their potential contribution to the pathogensis of CRC. Examples of these protagonists include *Streptococcus gallolyticus*, *Enterococcus faecalis*, *Escherichia coli*, *Fusobacterium* spp., and enterotoxigenic *Bacteroides fragilis* [12]. Formation of microbial biofilms in the colon can also alter the host tissue microenvironment and has been linked to colon carcinogenesis [13]. In addition, microbial metabolites, both the protective metabolites (e.g., acetate, propionate, butyrate) and detrimental metabolites (e.g., hydrogen sulfide, secondary bile acids) as well as the inflammatory signals elicited by those metabolites, are also suggested to be important contributors to CRC [14]. Furthermore, recent studies have presented strong evidence showing that commensal intestinal microbiota modulate the cancer response to therapy [15, 16], and interplay with the diet or dietary compounds on the CRC prevention and risk [17, 18].

In the aspect of CRC prevention, increasing evidences suggested that probiotics, prebiotics and synbiotics are conducive to CRC prevention [19]. Food fiber and phytochemicals such as polyphenols are considered as prebiotic dietary modifiers. They can influence the gut microbial communities, and in turn to modulate disease outcomes and drug responses of the host. Herbal saponins, a family of phytochemicals commonly found in many medicinal and dietary plants, have raised keen interest among scientists for their health-promoting effects, but have not been investigated for their potential intervention on gut microbiota. *Gynostemma pentaphyllum* (Gp) is rich in triterpenoid saponins and has been consumed as an herbal tea and as a folk medicine. Our recent studies indicated that saponins of Gp (GpS) enhanced beneficial intestinal bacteria and exerted prebiotics-like effects to the treated mice [20, 21]. We also showed that GpS effectively reduced grafted tumor in nude mice [21]. In this study, we presented further evidence of the effects of GpS on commensal microbial profile, intestinal environment, signaling molecules and mucosal cytokines in the CRC *Apc^Min/+^* mouse model. Alongside was the study on the cancer preventive effect of GpS. The findings from this work demonstrate that GpS remarkably improved the diseased intestinal epithelium of *Apc^Min/+^* mice through the modulation of the gut microbiota, downregulating the pro-inflammatory and upregulating the anti-inflammatory molecules. Such alteration may account for the cancer preventive activity of GpS observed in the treated animals.

## RESULTS

### Preventive treatment of GpS reduced the intestinal polyps in *Apc^Min/+^* mice

**Figure 1:**
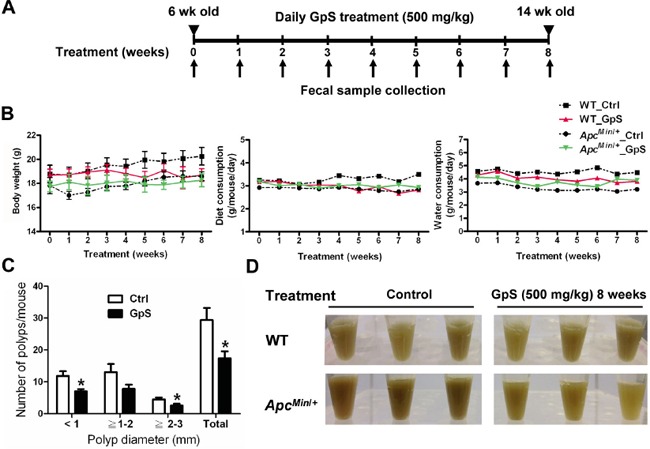
Effect of GpS on the intestinal polyp formation in the *Apc^Min/+^* mice **A.** Schematic diagram of experimental design. **B.** The profiles of body weight, diet and water consumption. **C.** Effect of GpS on the size distribution of polyps. Data is presented as the mean ± SEM (* P < 0.05 versus control); n=6/group. **D.** Display of the fecal extracts of the WT and *Apc^Min/+^* mice with or without GpS treatment for 8 weeks.

Normally, the intestinal polyps can be found in *Apc^Min/+^* mice at the age of 8-week in our hand. To investigate the preventive effects of GpS on polyp formation, the treatment was started on 6 weeks old mice. Single dose of GpS at 500 mg/kg or solvent control (0.5% CMC) was given daily by gavage for 8 weeks. The treatment scheme is illustrated in Figure 1A. Throughout the experimental period, none of the treated animals showed weight loss and abnormal food or water intake (Figure 1B). Figure 1C showed that administration of GpS significantly reduced the number of polyps by 40.68% (P < 0.05) when compared with the untreated controls. In the study, we found that the polyp formation in *Apc^Min/+^* mice was often accompanied with blood feces and darker color of the fecal extracts compared with their WT littermates. Interestingly, in the later stage of the treatment scheme, the fecal extracts again appeared in darker color in the untreated, but not in the treated *Apc^Min/+^* mice (Figure 1D).

### GpS treatment significantly altered the fecal microbiome of WT and *Apc^Min/+^* mice

**Figure 2:**
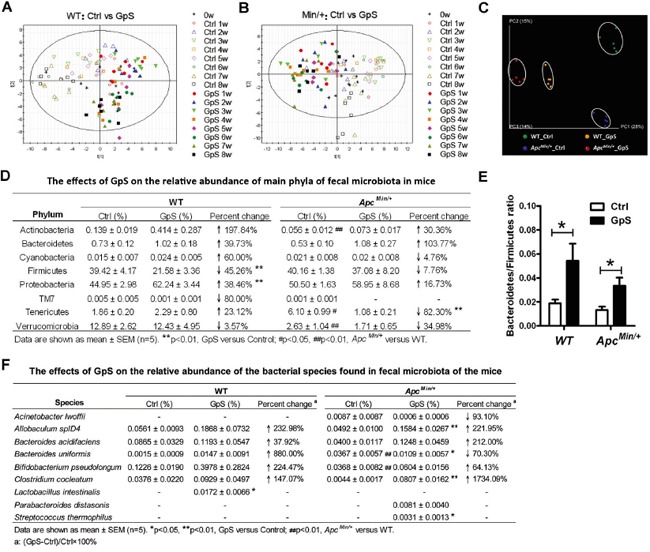
Comparison of microbial composition between the control and GpS-treated WT and *Apc^Min/+^* mice **A** & **B.** The time course PLS-DA plots of ERIC-PCR DNA profile of WT (see A) and *Apc^Min/+^* (see B) mice treated and untreated with GpS. Open symbols: control mice; Solid symbols: GpS-treated mice (n=6/group). Fecal genomic DNA was subjected to ERIC-PCR, and the gel pictures were digitized by Image Lab 3.0 system (Bio-Rad). Based on the distance and the intensity of each DNA bands, SIMCA-P 12.0 tool was applied to obtain the PLS-DA score plots. **C-E.** 16S pyrosequencing analysis on the fecal genomic DNA samples from the WT and *Apc^Min/+^* mice with or without GpS treatment for 8 weeks (n=5/group). C. PCoA plots of all samples from different treatment groups. The data were analyzed using QIIME software with the workflow script. PCoA plots were then generated using the unweighted UniFrac distance metric. D. The effects of GpS on the relative abundance of main phyla of fecal microbiota in mice. Beta diversity was calculated by QIIME software. E. *Bacteroidetes/Firmicutes* ratio. Data is presented as the mean ± SEM (* P < 0.05, GpS versus control). **F.** The effects of GpS on the relative abundance of the bacterial species found in fecal microbiota of the mice.

In order to understand the gut microbiota composition upon GpS treatment in *Apc^Min/+^* mice and their wild-type littermates, fecal samples were collected before the treatment, and weekly after the treatment for eight consecutive weeks (Figure 1A). The comparative study of microbial profiles between GpS-treated and untreated mice was conducted using ERIC-PCR analysis of the collected fecal samples. The resulting digitized data of ERIC-PCR fingerprints was analyzed by PLS-DA. Results showed a clear segregation of the microbial communities between the controls and GpS treated mice. This phenomenon existed in both the WT (Figure 2A) and *Apc^Min/+^* mice (Figure 2B).

To further examine the detailed composition of the fecal microbiome, 16S rRNA gene pyrosequencing was performed on the fecal DNA obtained from the WT and *Apc^Min/+^* mice at the W8 time point. Five fecal samples per group and a total of 20 samples were subjected for pyrosequencing. A total of 591,640 reads that passed quality control were produced with an average of 29,582 sequences per sample. PCoA plots showed a clear separation among the fecal microbiome of the four experimental groups (Figure 2C). The relative abundance of dominant phylum in the fecal microbiota also altered upon GpS treatment (Figure 2D). In the WT mice, GpS treatment markedly reduced the abundance of *Firmicutes* (from 39.42% down to 21.58%). In the meantime, it substantially increased the relative abundance of *Proteobacteria* (from 44.95% to 62.24%). In *Apc^Min/+^* mice, compared with the controls, mice treated with GpS exhibited relatively lower abundance of *Tenericutes* (from 6.10% down to 1.08%). In addition, in contrast to the untreated mice, the increased *Bacteroidetes*/*Firmicutes* ratio can be observed in both the GpS-treated *Apc^Min/+^* and WT mice (Figure 2E). Furthermore, the pyrosequencing data revealed that GpS altered the microbial communities at genus level (Supplementary Figure 1).

Pyrosequencing data also identified the increases of several well-known beneficial bacteria, including *Bacteroides acidifaciens, Bifidobacterium pseudolongum, Clostridium cocleatum*, *Lactobacillus intestinalis*, *Parabacteroides distasonis* and *Streptococcus thermophilus*. As shown in Figure 2F, compared with the WT, *Apc^Min/+^* mice showed a significant increase in the relative abundance of *Bacteroides uniformis*, but a substantial decrease in *Bifidobacterium pseudolongum*. However, GpS treatment restored the level of these two species to certain extent. Additionally, *Lactobacillus intestinalis* was unique to the GpS-treated WT mice. Compared with the *Apc^Min/+^* control mice, *Allobaculum spID4*, *Clostridium cocleatum* and *Streptococcus thermophilus* were significantly elevated upon GpS feeding. In addition, *Streptococcus thermophilus* and *Parabacteroides distasonis* were only detected in the GpS-treated *Apc^Min/+^* mice, and the relative abundance of *Bacteroides acidifaciens* was increased by 212.00% compared with the untreated *Apc^Min/+^* controls. It seemed that GpS treatment increased the levels of several bacterial species showing various beneficial effects to the host. In addition, certain potential opportunistic pathogen like *Acinetobacter lwoffii* [26] was only observed in the *Apc^Min/+^* mice and exhibited a 93.10% decrease upon GpS treatment.

### GpS treatment significantly reduced sulfate-reducing bacteria in *Apc^Min/+^* mice

**Figure 3:**
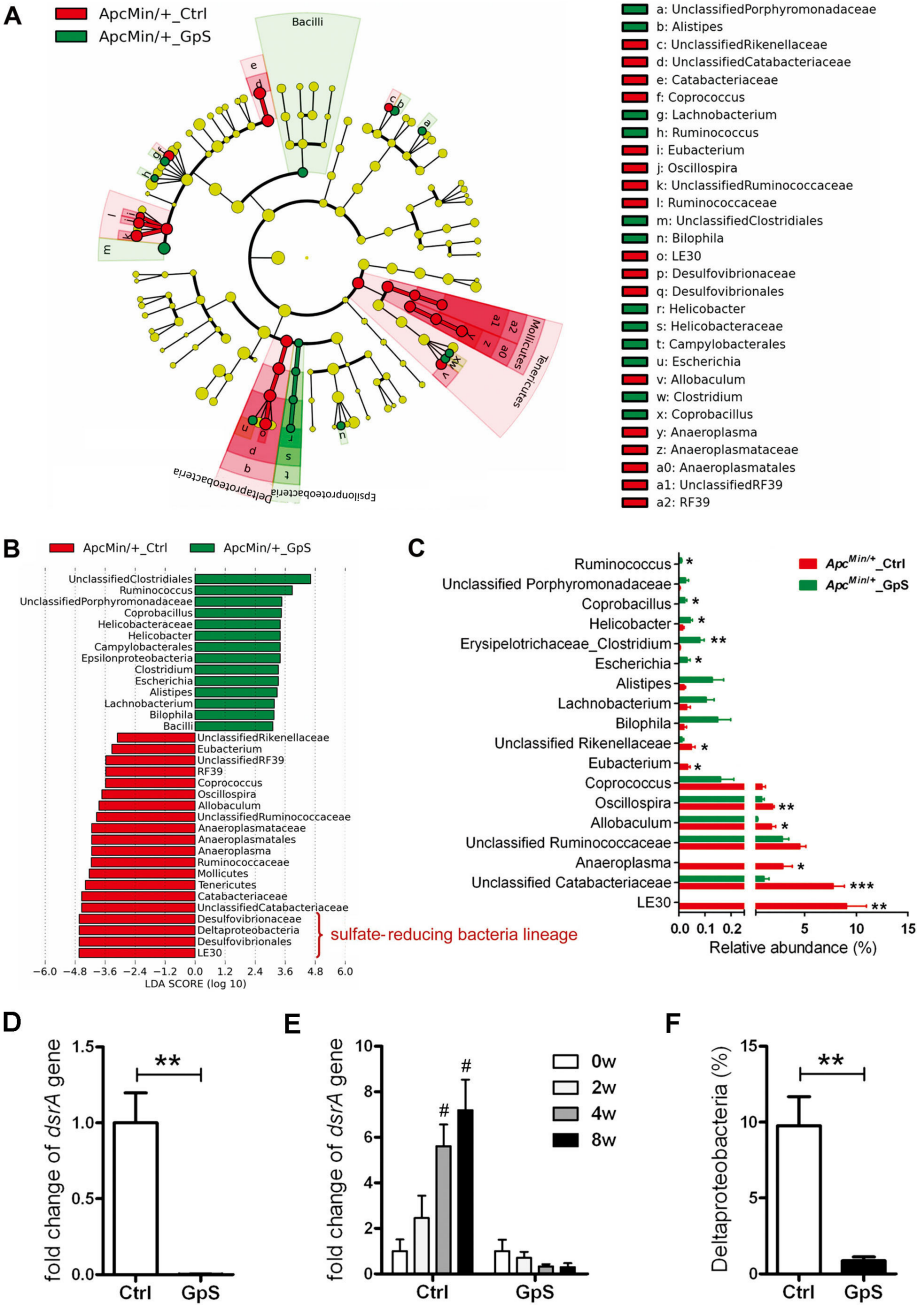
Identification of the key phylotypes in the fecal microbiome of GpS-treated and untreated *Apc^Min/+^* mice **A.** Taxonomic representations of the fecal microbiome. The differentially abundant taxa are presented with different colors using LEfSe method. The taxa from the untreated and GpS-treated *Apc^Min/+^* mice are colored in red and green, respectively. The taxa with non-significant changes are colored in yellow. Each circle's diameter represents the taxon abundance. **B.** Histogram of the LDA scores of fecal 16S rRNA gene sequences of the untreated controls (red color) and GpS-treated *Apc^Min/+^* mice (green color). LDA scores characterized the magnitude of differential abundance in the microbial taxa between compared samples. **C.** The relative abundance of differentially abundant genera. Data is presented as the mean ± SEM (* P < 0.05, ** P < 0.01, *** P < 0.001, GpS versus control); n=5/group. **D.** Fold change of dissimilatory (bi)sulfite reductase (*dsrA*) gene in fecal genomic DNA samples obtained from mice treated with GpS for 8 weeks. The DNA subjected to qRT-PCR here was the same as the one applied to pyrosequencing. The same amount of DNA was used as template, and the level of *dsrA* gene was normalized to 16S rRNA gene. 16S rRNA gene is a segment of prokaryotic DNA found in all bacteria, and a universal primer set was used to detect the 16S rRNA gene of total bacteria. **E.** Time course of relative expression of *dsrA* gene. qRT-PCR was used to determine the level of *dsrA* gene and normalized to that of the total fecal bacteria, and expressed as fold change of the WT control group (see D) or fold change over the 0w sample (before treatment) of each mouse (see E). **F.** Relative abundance of *Deltaproteobacteria*. Data is presented as the mean ± SEM (** P < 0.01 GpS versus control samples; ^#^ P < 0.05 versus 0w samples); n=6/group.

We then further investigated the key phylotypes responsible for the differences of fecal microbiome by LefSe tool. Within the *Apc^Min/+^* mice group, four lineages were identified as the main contributors to the differences in the fecal microbiome structure between GpS-treated and untreated *Apc^Min/+^* mice. Three lineages, including *Deltaproteobacteria-Desulfovibrionales-Desulfovibrionaceae-LE30*, *Tenericutes-Mollicutes-RF39*-Unclassified *RF39*, and *Tenericutes*-*Mollicutes*-*Anaeroplasmatales*-*Anaeroplasmataceae-Anaeroplasma*, were overrepresented in the untreated *Apc^Min/+^* mice, whereas *Epsilonproteobacteria-Campylobacterales-Helicobacteraceae-Helicobacter* lineage was relatively enriched in the GpS-treated *Apc^Min/+^* mice (Figure 3A). It was noteworthy that genus *LE30*, affiliated with the sulfate-reducing bacteria (SRB) family *Desulfovibrionacea*, was identified with a very high LDA score (Figure 3B), reflecting marked abundance in *Apc^Min/+^* control mice. Interestingly, *LE30* was completely depleted in the GpS-treated *Apc^Min/+^* mice. Likewise, *Anaeroplasma* and *Eubacterium* were also absent from the GpS-treated individuals. Conversely, *Ruminococcus*, *Coprobacillus* and *Escherichia* were unique to the GpS-treated *Apc^Min/+^* mice. All these unique genera showed statistically significant difference in the relative abundance between the GpS-treated and untreated *Apc^Min/+^* mice (Figure 3C). Most *Helicobacter* and *Escherichia* are commensal gut microbiota, while particular strains are pathogenic. In our study, none of the known pathogenic microbes were observed in either treated or untreated mice, although GpS-fed *Apc^Min/+^* mice showed higher relative abundance of *Helicobacter* and *Escherichia* at the genus level.

Sulfate-reducing bacteria are able to reduce sulfate to hydrogen sulfide (H_2_S) through a process termed “dissimilatory sulfate reduction”. Hydrogen sulfide can damage the intestinal epithelium leading to chronic inflammation and imbalance between cellular proliferation and apoptosis, indicating a possible association of SRB with CRC [22, 23]. The dissimilatory (bi)sulfite reductase (*dsrA*) gene, a crucial gene of SRB, is involved in the energy metabolism of SRB and have been employed as a reliable marker for the presence of SRB [24]. We then performed real-time qRT-PCR of the *dsrA* gene to quantify SRB in the W8-fecal samples. Compared with the controls, significant down-regulation of *dsrA* was observed in the fecal DNA samples of GpS-treated *Apc^Min/+^* mice (Figure 3D), which is in line with the data of decreased sulfate-reducing bacteria lineage obtained by pyrosequencing analysis (Figure 3B). We also performed qRT-PCR of *dsrA* in fecal samples collected at different experimental time points. The level of *dsrA* increased in the control, while decreased in the GpS-treated *Apc^Min/+^* in a time-dependent manner. These finding indicated that there might be a correlation between polyp development and sulfate-reducing bacteria abundancy (Figure 3E). *Deltaproteobacteria* is one of the major phylogenetic lineages of SRB [25]. Compared with the controls, GpS-treated *Apc^Min/+^* mice showed a substantial reduction in the relative abundance of *Deltaproteobacteria* as validated by pyrosequencing analysis (Figure 3F). These findings suggest a suppressing effect of GpS on sulfate-reducing bacteria, for which the polyp formation was reduced.

### GpS treatment improved the intestinal epithelial barrier of *Apc^Min/+^* mice

**Figure 4:**
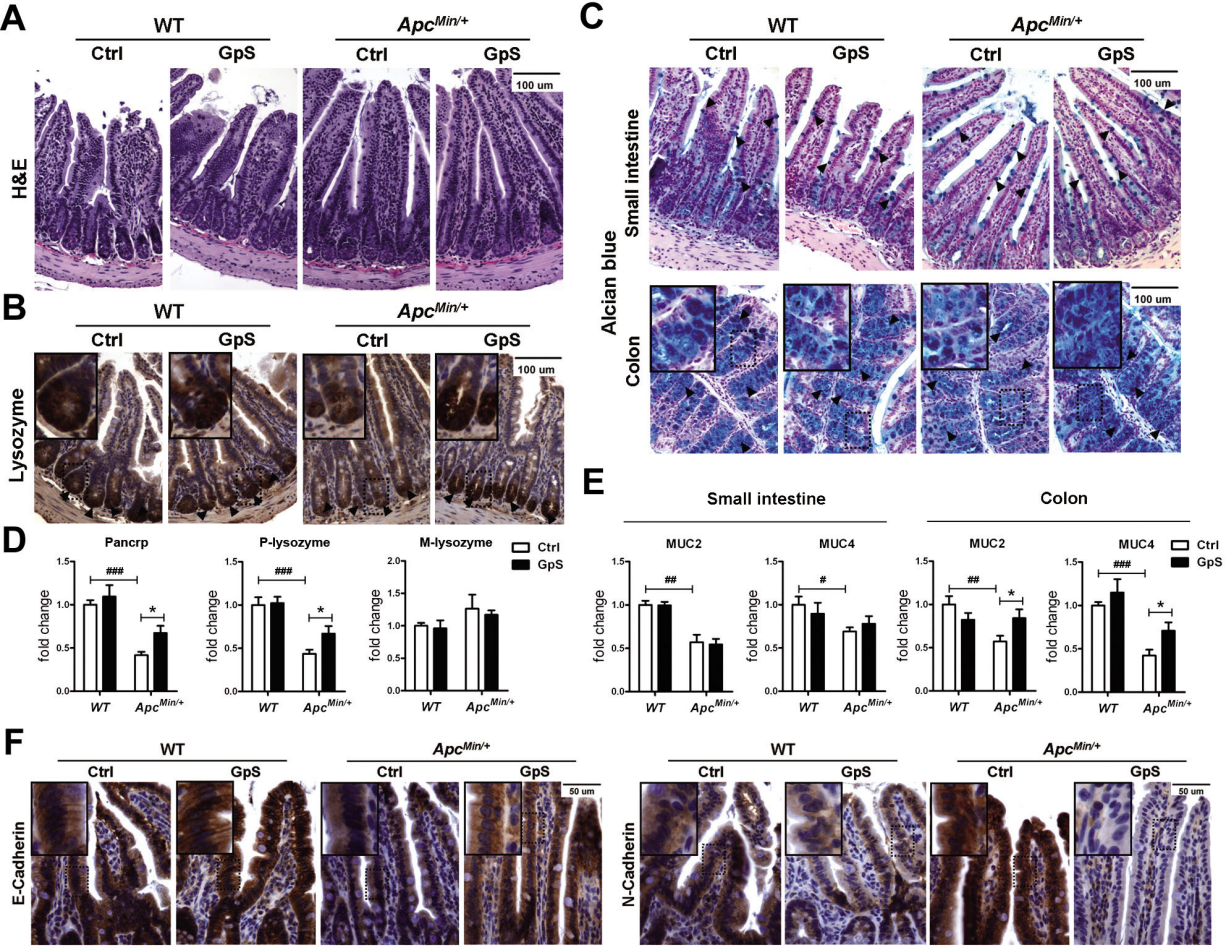
Effects of GpS on the intestinal epithelium Intestinal tissues were collected after 8 weeks of treatment with or without GpS from the WT and *Apc^Min/+^* mice. **A.** H&E staining. **B.** IHC staining of Paneth cells. **C.** Alcian blue staining of goblet cells. Hematoxylin and eosin (H&E) staining was used to visualize the formalin-fixed sections of small intestine. IHC staining of lysozyme was applied to identify the Paneth cells in the small intestine, and the dark brown at the bottom of the intestinal crypts indicates the presence of Paneth cells. Alcian blue staining was used to identify the goblet cells, and the blue staining indicates the presence of the goblet cells. **D & E.** The relative mRNA expression of Paneth cells related antimicrobial peptide (see D) and goblet cells related mucins (see E) was evaluated by qRT-PCR in the intestinal mucosal samples. Data was normalized to the expression of reference gene, and expressed as fold change of the WT control group. Data is presented as the mean ± SEM (* P < 0.05, GpS versus control samples; ^##^ P < 0.01, ^###^ P < 0.001, *Apc^Min/+^* versus WT control samples); n=6/group. **F.** IHC staining of E-cadherin and N-cadherin. Positive expression is indicated by the brown color staining. Nuclear is stained and appeared in blue color that was done by hematoxylin staining.

Gut epithelial barrier dysfunction of *Apc^Min^*^/*+*^ mice has been reported in several studies, and such defective epithelial barrier can facilitate the translocation of inflammatory cytokines, resulting in the promotion of tumor growth [4, 27]. After revealing the impact of GpS on the gut microbiota of WT and *Apc^Min/+^* mice, we investigated any corresponding changes of the epithelium under GpS treatment. Paneth cells, along with goblet cells, enterocytes, and enteroendocrine cells, are the principal cell types of the intestinal epithelium. We first examined the general intestinal morphology by H&E staining and observed no obvious difference between the control and GpS treatment groups (Figure 4A). Paneth cells, which are normally located at the bottom of the crypts in the small intestine, are a principal source of antimicrobial substances, including lysozyme and α-defensins. Immunohistochemistry (IHC) staining for lysozyme, which is used as the marker for the presence of Paneth cells, demonstrated a reduction of Paneth cells in the *Apc^Min/+^* mice compared with their WT littermates (Figure 4B). Goblet cells take responsibility for generating mucus, which constitutes the first line of immune defense. The result indicated a decrease of goblet cells in the *Apc^Min/+^* mice compared to the WT mice, particularly in the colonic region (Figure 4C). Interestingly, the lysozyme-expressing Paneth cells and Alcian blue positive goblet cells in the GpS-treated *Apc^Min/+^* mice was comparable to the WT mice. Consistent results were obtained by examining the mRNA expressions of microbicidal peptide and mucins secreted by Paneth cells and goblet cell using qRT-PCR. The mRNA of α-defensins (Pancrp), P-lysozyme, MUC2 and MUC4 were significantly reduced in the *Apc^Min/+^* mice relative to the WT controls. However, GpS treatment tended to restore the mRNA levels of α-defensins and P-lysozyme. In mice, there are two common forms of lysozyme. The P lysozyme is expressed in intestinal epithelium, especially in the Paneth cells. The M lysozyme is expressed mainly in myeloid cells. In our study, no differences were observed in M-lysozyme expression among different experimental groups (Figure 4D). Likewise, the mRNA levels of MUC2 and MUC4 in the colon tissue were highly expressed in the GpS-treated *Apc^Min/+^* mice than the controls (Figure 4E). These data suggested that GpS treatment might improve the intestinal epithelial barrier in the *Apc^Min/+^* mice by increasing the number and secretions of Paneth and goblet cells.

E-cadherin is not only a key adherens junction molecule, it is also required for intestinal morphogenesis, and Paneth cell maturation among other functions [28]. Impaired expression of E-cadherin has been linked to defective gut barrier function [29], and switching expression from E-cadherin to N-cadherin was found to be associated with CRC progression [30]. We therefore examined the expressions of E-cadherin and N-cadherin in the small intestines by IHC staining. In comparison to the WT mice, an obvious decrease in E-cadherin and increase in N-cadherin were observed in the small intestines of the *Apc^Min/+^* mice. GpS treatment effectively reversed the trend, for which the level of E-cadherin was up-regulated and N-cadherin was significantly down-regulated (Figure 4F), showing an improvement of the pathological condition of the intestinal epithelium.

### GpS down-regulated protein expressions of p-Src, p-STAT3 and β-catenin in intestinal mucosa

**Figure 5:**
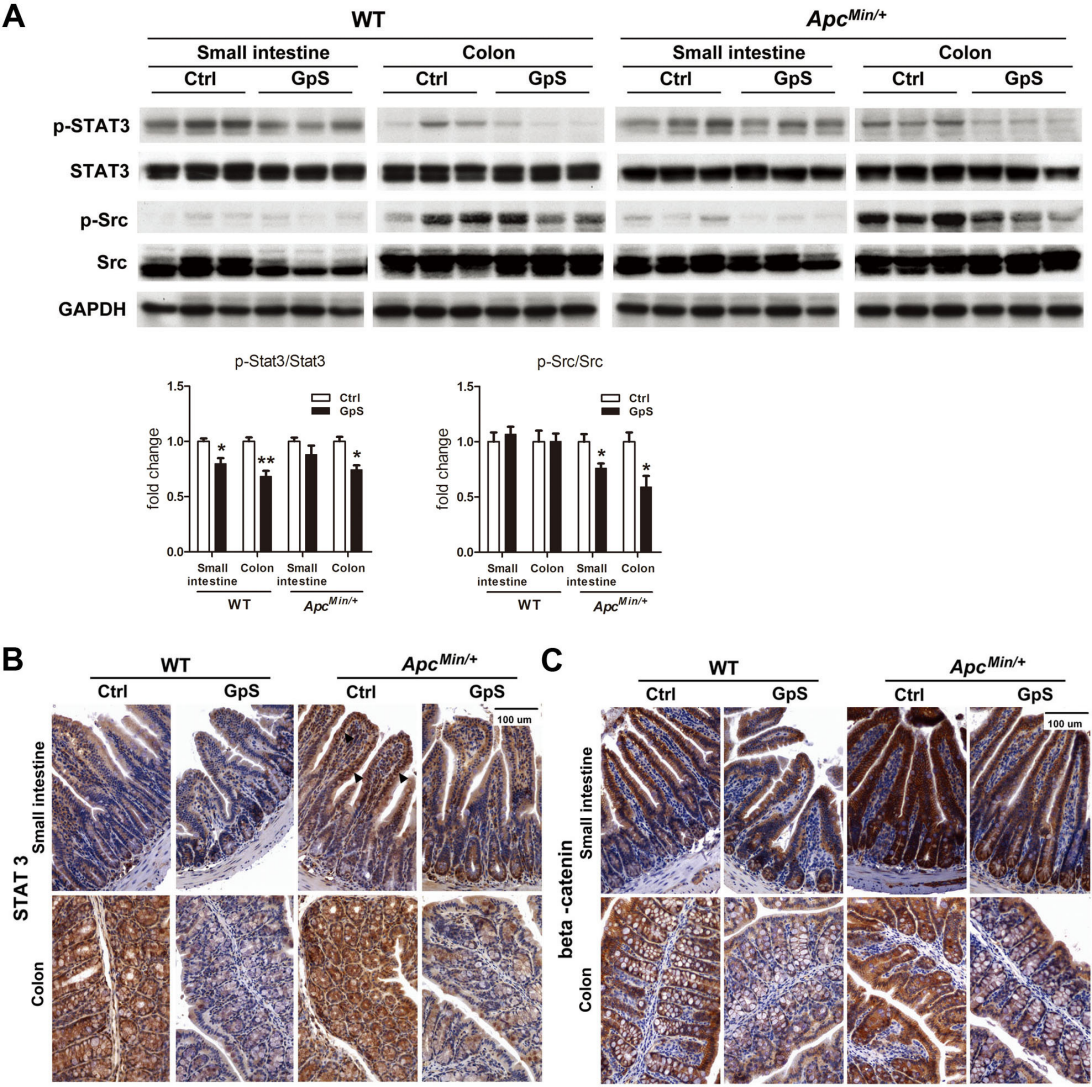
Effects of GpS on the protein expression of STAT3 and beta-catenin **A.** Western blot analysis: mucosa from the small intestine and colon were collected after 8 weeks of treatment. Mucosal protein lysates were analyzed by western blotting with specified indicated antibody. GAPDH was used as a loading control. Each lane represents sample obtained from individual mouse (n=3/group). **B & C.** IHC staining of STAT3 (see B) and beta-catenin (see C) in the small intestine and colon. Arrows indicate the STAT3 nuclear translocation.

Signal transducer and activator of transcription 3 (STAT3) can negatively regulate E-cadherin and positively modulate N-cadherin [31] and has become a promising target for cancer immunotherapy [32]. The gut microbiota has also been shown to enhance tumor burden in *Apc^Min/+^* mice partially via STAT3 phosphorylation [4]. Aberrant β-catenin expression is known to be involved in CRC development, and the resident intestinal bacteria is associated with the stability of β-catenin in intestinal epithelial cells [33]. We thus investigated the impact of GpS on β-catenin, phosphorylation of STAT3 (p-STAT3) and the STAT3 activator, phosphorylated Src (p-Src) proteins in the intestine. Here, we found that GpS treatment down-regulated p-STAT3 and p-Src, in particular in the colonic mucosa in the western blotting analysis (Figure 5A). As shown in Figure 5B, nuclear STAT3 was observed in the small intestine of the *Apc^Min/+^* mice, but hardly appeared in the nuclei of the GpS-treated epithelial cells. These results are consistent with the down-regulated effect of GpS on the p-STAT3 that is required for nuclear translocation of the protein. IHC staining also further revealed the down-regulated effect of GpS on the expression of β-catenin (Figure 5C). Altogether, we revealed that GpS treatment increased E-cadherin but decreased N-cadherin in the *Apc^Min/+^* mice, and the down-regulation of p-STAT3 might account for such results.

### GpS might facilitate polarization of M2 macrophage and improve the intestinal barrier

**Figure 6:**
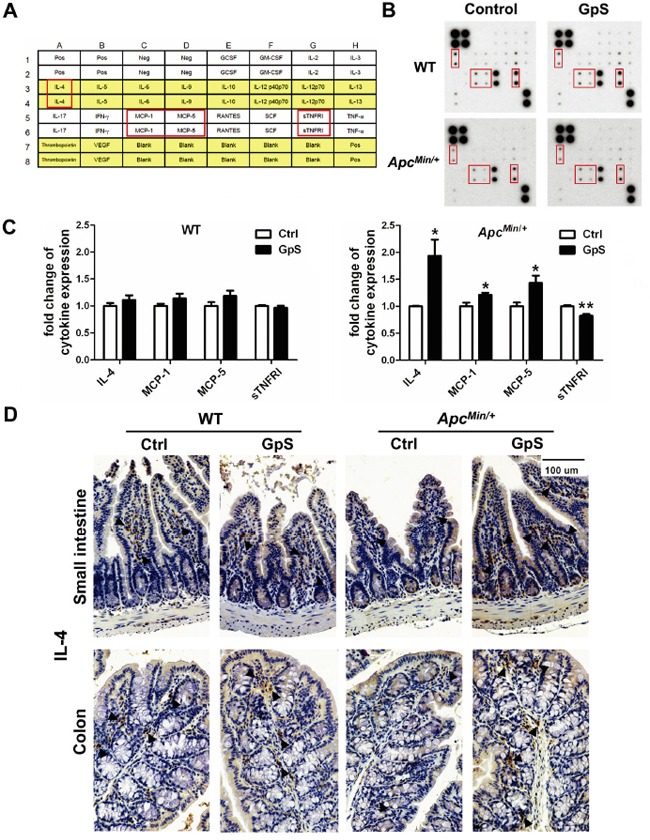
Effects of GpS on the mucosal cytokine profiles Mucosal lysates from five selected mice per group were pooled together, and analyzed using the cytokine array kit. **A.** The location of detected cytokines in the membrane. **B.** Representative cytokine array blots showing differential expressed cytokines. **C.** Densitometric analysis of the altered cytokines upon GpS treatment. Data was normalized to the positive control and presented as fold changes relative to the controls. Results were representative of two independent experiments with duplicate in each membrane. Data is presented as the mean ± SEM (* P < 0.05, ** P < 0.01, GpS versus control group). MCP: monocyte chemoattractant protein; sTNFRI: soluble tumor necrosis factor receptor I. **D.** IHC staining of IL-4 in the small intestine and colon. Arrows indicate the representative staining of the positive cells.

Cytokines have been suggested to play a crucial role in regulating immune response between the mucosal barrier and the commensal microbiota. To investigate the effect of GpS treatment on cytokine profiles, RayBiotech mouse cytokine array containing 22 main cytokines (Figure 6A) was used to detect the cytokines in the intestinal mucosal protein from experimental groups (Figure 6B). We observed that the levels of IL-4, MCP-1 and MCP-5 were significantly increased, whereas sTNFRI was significantly decreased in the intestinal mucosa from GpS-treated *Apc^Min/+^* mice compared with the controls. However, the effect of GpS treatment was not apparent in the WT (Figure 6C). IHC staining of IL-4 further confirmed the finding in the cytokine array (Figure 6D). IL-4 has been reported to induce mucin secretion in goblet cells [34], which echoed our earlier results showing concurrent elevations of IL-4 and mucins were observed in the GpS-treated *Apc^Min/+^* mice in contrast to the controls.

**Figure 7:**
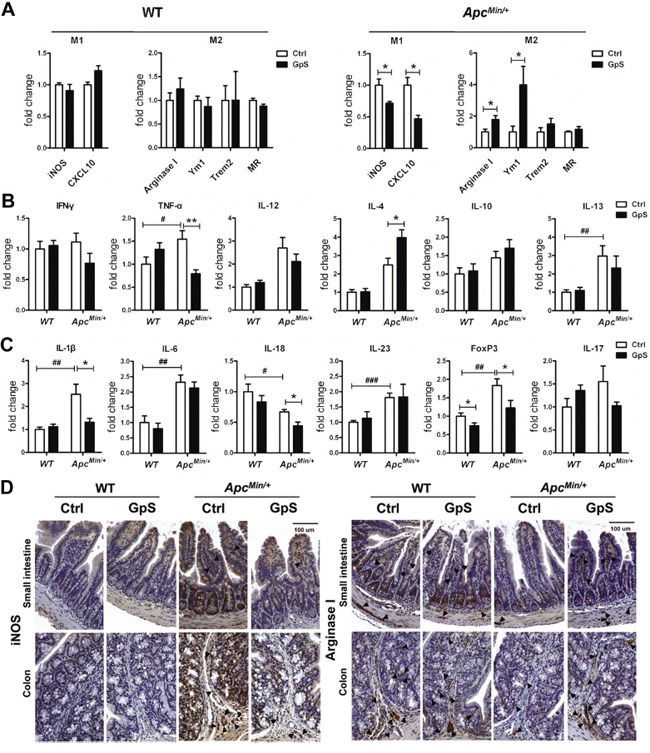
Effects of GpS on the macrophage phenotypic polarization **A.** The relative mRNA expression of M1 and M2 macrophage markers. qRT-PCR analysis of mRNA extracted from the mucosal lysates of experimental mice were performed with specific primers. Data was normalized to the expression of reference gene, and expressed as fold change of the untreated group. **B & C.** The relative mRNA expression of (see B) macrophage polarization related cytokines and (see C) inflammation related molecules. Data was normalized to the expression of reference gene, and expressed as fold change relative to the WT control group. Data is presented as the mean ± SEM (* P < 0.05, ** P < 0.01 GpS versus control samples; ^#^ P < 0.05, ^##^ P < 0.01, ^###^ P < 0.001, *Apc^Min/+^* versus WT control samples); n=6/group. **D.** IHC staining of iNOS and Arginase I. Arrows indicate the representative staining of the positive cells.

IL-4 is the stimulus for alternatively activated M2 macrophages whose primary roles are in tissue repair and anti-inflammation [35, 36]. To evaluate the phenotype of macrophages in the intestine, we next investigated the mRNA expressions of several M1 and M2 markers by qRT-PCR. In the *Apc^Min/+^* mice, mRNA of iNOS and CXCL10, which are the key effector molecules produced by pro-inflammatory M1 phenotype, were significantly lower in the normal mucosa of the GpS-treated mice compared with the controls. On the other hand, expressions of arginase 1, Ym1, Trem2 and MR, which are the typical anti-inflammatory M2 phenotypes markers, were higher in the GpS-treated *Apc^Min/+^* mice than the untreated controls. Differences were not observed between the GpS-treated and untreated WT mice (Figure 7A).

IFN-γ, TNF-α and IL-12 are responsible for inducing M1 phenotype, while IL-4, IL-10 and IL-13 are M2-polarizing cytokines. Since macrophages can alter their phenotype in response to the microenvironment where they exist, we further investigated those factors that can affect the polarization of macrophages. Compared with the WT mice, the mRNA expression of TNF-α and IL-13 were elevated in the *Apc^Min/+^* mice. Interestingly, the mRNA expression of TNF-α was lower, while IL-4 was higher in the intestinal mucosa of the GpS-treated *Apc^Min/+^* mice relative to the untreated controls (Figure 7B). Thus, it seemed that the cytokine expression profile of intestinal microenvironment was consistent with the increased M2 macrophage phenotype. Subsequent evaluation of molecules in relation to inflammatory response revealed that except IL-18, the mRNA levels of IL-1β, IL-6, IL-23, FoxP3 and IL-17 were significantly elevated or showed an increasing trend in the *Apc^Min/+^* mice compared with the WT (Figure 7C). These findings demonstrated a high inflammatory status in the intestinal mucosa of the *Apc^Min/+^* mice. Remarkably, after GpS feeding, the *Apc^Min/+^* mice showed a significant decrease in the mRNA expression of IL-1β, IL-18 and FoxP3 without obviously affecting IL-6, IL-23 and IL-17. Chronic inflammation of the intestinal mucosa is associated with an increased risk of developing CRC. These data supported the role of GpS in the process of inflammation during intestinal tumorigenesis.

To further substantiate our findings regarding repairing effects of GpS on the intestinal epithelium, we next applied IHC staining to examine the expression of macrophage subtype markers in the intestinal mucosa to further confirm the polarizing effects of GpS treatment on macrophages. iNOS and Arginase I are the common markers for M1 and M2 macrophages, respectively [37]. In contrast to the WT mice, the *Apc^Min/+^* mice exhibited relatively higher expression of iNOS and lower expression of Arginase I. Meanwhile, GpS-treated *Apc^Min/+^* mice demonstrated increased Arginase I and decreased iNOS immunoreactivity compared with the controls (Figure 7D). Collectively, these findings indicated that GpS treatment might alter cytokine profile by enhancing IL-4 and thus skewed M1 macrophages to M2 phenotype in the intestinal mucosa microenvironment, contributing to the intestinal tissue repair.

## DISCUSSION

Currently, little is known about the function of herbal saponins in the homeostasis of the intestinal microenvironment. This study is conducted to test the hypothesis that dietary GpS supplements may alter intestinal microbiota and mucosal barrier of the host, thereby impact on the cancer preventive function of Gp saponins. In this study employing a colonic carcinogenic *Apc^Min/+^* mouse model, we set forth to investigate the GpS effects on the growth of tumor in the gut; the composition of fecal microbiota; the host's intestinal mucosal barrier; and the intestinal inflammation status of host. To our best knowledge, the results presented here are the first in-depth study to demonstrate a novel role of botanical saponins in the homeostasis of gut microbiota and mucosal environment.

In the study, we found that the WT and *Apc^Min/+^* mice exhibited similar profile of fecal microbiome at 6 wk of age. As the mice grew older, disparity of microbial profiles between the WT and their Min/+ littermates become apparent (Supplementary Figure 2). This is coincided with the timing of onset of intestinal polyps which usually starts in 8 to 10 wk of age [38] In our recent study [21], we also demonstrated that tumor grafting can significantly alter the composition of gut microbiota in nude mice. Based on these observations, it suggests that tumor growth could impact the gut microbiota, whether the tumors form in the gut, or distant from the gut. On the other hand, we are not rule out that the aberrant Wnt/β-catenin signaling alone could affect the composition of gut microbiota in *Apc^Min/+^* mice, and it is an interesting area to be explored. Asides from the above findings, one of the key findings of this study is the prebiotic-like effect of GpS by which a favorable growth environment was tuned for the propagation of beneficial microbes. For instance, GpS increased the abundance of *Bifidobacterium pseudolongum*, which is a beneficial inhabitant in the intestine and known as probiotics [39]. *Streptococcus thermophilus* is an essential lactic acid bacterium, and commonly used in the production of yogurt [40]. Oral administration of bacterial components derived from *Parabacteroides distasonis* was reported to reduce chronic intestinal inflammation [41]. Interestingly, both *Streptococcus thermophilus* and *Parabacteroides distasonis* can only be detected in the GpS-treated *Apc^Min/+^* mice. GpS treatment stimulated *Clostridium cocleatum*, for which the colonization of harmful bacteria *C. difficile* was prevented and decrease of intestinal diseases was reported [42, 43]. Additionally, an increasing trend of *Bacteroides acidifaciens* and elevated transcripts of IgA transport related J-chain gene (Supplementary Figure 3) were observed in the GpS-fed mice, which coincided with previous reports showing that *Bacteroides acidifaciens* can promote IgA production, which may contribute to maintain the intestinal homeostasis [44].

More importantly, besides upregulating the beneficial bacteria, we also demonstrated that the abundance of sulfate-reducing bacteria linkage as well as the critical enzyme dsrA for H_2_S production in SRB decreased significantly in the *Apc^Min/+^* mice fed with dietary Gp saponins. SRB, such as *Desulfovibrio* and *Bilophila*, are common colonic inhabitants found both in humans and mice. They produce and use H_2_S for energy harvesting in the gastrointestinal track and have been found to be associated with gastrointestinal diseases and cancer [45]. Expansion of SRB, such as *Bilophila wadsworthia*, has been found in hosts that are genetically susceptible or have impaired function of mucosal barrier [46]. The presence of H_2_S has been suggested as a potential etiological agent in gastrointestinal disease due to its genotoxic, cytotoxic and inflammatory effects [47]. Study also showed that H_2_S can contribute to the cancer progression trigged by the genotoxic insult to the colonic epithelium [48]. Higher level of H_2_S has been reported in the stool of individuals with high risk of CRC [49]. Moreover, hydrogen sulfide-producer *Fusobacterium nucleatum* has been found to be associated with colorectal cancer [50, 51]. Interestingly, recent report showed that the prebiotics treatment in mice was able to decrease the population of a group of SRB which was significantly elevated in mice under treatment of high fat diet [52]. Their findings are in line with our current observation with GpS treatment. In our recent study, we demonstrated that Gp saponins along with few other saponins from edible plants do exhibit prebiotic-like properties [20]. Here, we postulate that the profound effect of GpS on the reduction of SRB lineage may alleviate the deleterious effects evolved by the growth of intestinal tumor in the *Apc^Min/+^* mice and improve the host gut barrier. Considering the fact that colonic mucosa is persistently colonized by SRB, the beneficial versus toxic effects of H_2_S need to be elucidated.

Intestinal epithelial cells consisted different cell types, out of which are the anti-microbial peptides-secreted Paneth and mucin-secreted goblet cells. Paneth cells play an important role in maintenance of host-microorganism homeostasis in small intestine, while goblet cells contribute to innate immune defense. Lack of mucin can lead to a defective mucus barrier and result in increased pathogenic bacterial adhesion and penetration into surface epithelial cells, and increase intestinal permeability [53]. Study showed that Paneth cell dysfunction can affect the secretion of α-defensins and cause the microbial imbalance, predisposing the host to intestinal inflammation [54]. α-defensin deficiency can also cause a decrease in the relative abundance of *Bacteroidetes* but an increase in *Firmicutes* [55]. In our case, compared with the control mice, GpS-treated *Apc^Min/+^* mice showed increased population of Paneth cells in the small intestine and also displayed increased ratio of *Bacteroidetes*/*Firmicutes*. Furthermore, E-cadherin, which was found strikingly upregulated in GpS-treated *Apc^Min/+^*, is also playing a key role in the maturation of Paneth and goblet cells [29]. Overexpression of STAT3 has been shown to dramatically downregulate E-cadherin and upregulate N-cadherin in CRC cells and to lead to CRC cells invasion and resist to apoptosis [31]. Here, we found daily feeding of GpS supplements effectively downregulated p-STAT3 in the treated Min/+ mice. In the same group of mice, we detected a marked increase in E-cadherin and decrease in N-cadherin. Collectively, these findings provide a strong evidence to support the cancer preventive property of GpS.

IL-4 is known as an anti-inflammatory and as an immunoregulator cytokine. Early reports showed that defect in IL-4 expression in the colonic mucosa was closely associated with patients suffered from inflammatory bowel disease (IBD) and the risk of CRC [56, 57]. IL-4 was suggested to inhibit colon cancer cell growth [58]. Thus, a continuous expression of IL-4 may provide an effective therapy for various diseases, including cancers and immunologic disorders [59]. IL-4 has been reported to induce mucin secretion in goblet cells [34]. It can also promote alternative activation of macrophages into M2 cells, and increase of M2 cells can contribute to an enhanced tissue repair and reduce pathological inflammation [35, 36]. Therefore, we supposed that the GpS on the protective effects in the gut mucosal barrier might be partially through the induction of IL-4 secretion, as well as the polarization of M2 macrophages. The function of the macrophage subtypes in normal tissue is known to be quite different from that of tumor associated macrophages (TAMs). In this study, we only focused on the polarizing effects of GpS on the macrophages within intestinal mucosa to elucidate the gut barrier function. Such effects may be different if in the pathological situations like TAMs of local polyps. All these possibilities remain to be investigated in the future.

In addition to IL-4, GpS significantly increased MCP-1 and MCP-5 that possess tumoricidal activity of macrophages *in vivo*. MCP-1 can recruit monocytes, T-lymphocytes and dendritic cells to the inflammatory sites of tissue injury or infection [60, 61], and IL-4 functions as a potent stimulator for MCP-1 expression [62]. Recent studies have revealed that MCP-1 is mainly produced by goblet and Paneth cells [63]. We postulate that the increased IL-4 and the increase population of Paneth cells and goblet cells induced by GpS might account for the elevation of MCP-1 and/or MCP-5 in our study. More interestingly, GpS-treated *Apc^Min/+^* mice showed a reduction in *Coprococcus* and an increase of mucosal MCP-1, as increased MCP-1 has been reported to be negatively correlated with the abundance of *Coprococcus* [64].

**Figure 8:**
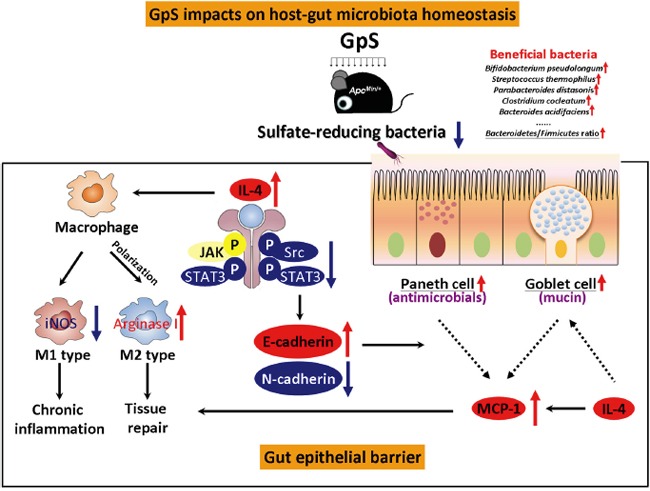
Summary of the impacts of GpS on host-gut-microbiota in *Apc^Min/+^* mice GpS impacts on host-gut-microbiota homeostasis through various means. GpS treatment increases beneficial bacteria, decreases sulfate-reducing bacteria, and improves gut epithelial barrier, which might contribute to its cancer preventive effects. The protective effects of GpS on the gut epithelial barrier (box in solid lines) might be partially through the induction of IL-4 secretion. On one hand, the elevation of IL-4 stimulates the M1 to M2 macrophages switching and facilitates intestinal tissue repair. On the other hand, IL-4 expression can also suppress the M1 induced marker, iNOS and reduce the chronic inflammation in the gut epithelial barrier. The elevated IL-4 cytokine might account for downregulation of p-Src and p-STAT3. As a result, it positively regulates E-cadherin and negatively modulates N-cadherin, presenting a reversion of disease to health status of gut epithelium upon GpS treatment. GpS also seems to improve of the intestinal epithelium by increasing Paneth and goblet cells. E-cadherin is required for Paneth cell maturation, while IL-4 can induce mucin secretion in goblet cells. The enrichment of goblet and Paneth cells facilitates the MCP-1 production and contributes to the tissue repair. Black arrows: pathways; red arrows: up-regulation; blue arrows: down-regulation; dashed lines: increase production.

In summary, this study provides a unique insight into the intricate interplay between the host and gut microbiota upon dietary herbal saponins administration. Here we show that GpS effectively enhance beneficial commensal bacteria, and substantially reduce the sulfate-reducing bacteria. To the host intestinal epithelial barrier, GpS remarkably suppress a repertoire of pro-inflammatory, and pro-oncogenic cytokines and signaling molecules, and present an overall anti-inflammatory and anti-oncogenic epithelial microenvironment in the gut of Apc^*Min/+*^ mice. Thus, we propose that GpS may play an crucial role in bringing the disease state of the host to a balance and healthy state through the modulation of the interaction between host and gut microbiota, which may contribute to its preventive efficacy against the tumorigenesis in *Apc^Min/+^* mice (Figure 8). These findings provide first hand evidence for the impact of herbal saponins on the gut microbial ecosystem and new insights into the possible mechanisms for their cancer preventive effects. Such health beneficial effects of GpS may apply to alleviate other chronic elements associated with inflammatory intestinal environment.

## MATERIALS AND METHODS

### Animals and treatments

Experimental procedures were conducted according to the guidelines for the care and use of laboratory animals. All procedures were approved by the Baptist University Ethics Review Committee for animal research. Heterozygous male *Apc^Min^*^/*+*^ (C57BL/6J-*Apc^Min^*^/*+*^) and female wild-type (WT) C57BL/6J mice were purchased from Jackson Laboratory. An in-house breeding colony has been maintained by breeding C57BL/6J-*Apc^Min^*^/*+*^ male to WT female C57BL/6J mice. The *Apc^Min^*^/*+*^ genotype of offspring is confirmed by polymerase chain reaction analysis. Mice were fed with PicoLab^®^ Rodent Diet 20-5053 (LabDiet, USA), and housed in a 12-h light/12-h dark cycle facility with free access to food and water. GpS was purchased from Hauduo Natural Products (Guangzhou, China). According to the procedures outlined by Wu *et al*. [65], each batch of GpS was authenticated and chemically profiled. GpS was dissolved in 0.5% carboxymethyl cellulose (CMC) at 50 mg/ml. Single dose of GpS at 500 mg/kg or solvent control was given daily by gavage, started at 6 weeks of age before the appearance of spontaneous intestinal polyps of the animals. The treatment was carried out for 8 weeks. Total twelve female mice were used for each experimental group, including WT-control, WT-GpS, *Apc^Min^*^/*+*^-control, and *Apc^Min^*^/*+*^-GpS groups. Six mice per group were used in the first batch of experiment, followed by three mice per group were used in the second and third batches of experiment, respectively. The second and third batches of experiment were applied to collect more intestinal mucosa from the experimental mice for the subsequent experiments. The mice with the same genotype and the same treatment were housed in the same cage for the first batch of experiment, while mice with the same genotype but different treatment (GpS-treated or untreated) were co-housed in the same cage for the second and third batches of experiment. Euthanasia of animals was carried out according to the guidance of the American Veterinary Medical Association (AVMA). Total 48 mice were used in this study, and carbon dioxide (CO_2_) inhalation was used for euthanasia of mice.

### Fecal samples collection and bacterial genomic DNA extraction

Fecal samples were collected from each mouse for two consecutive hours from 8:00 to 10:00 A.M. before treatment and weekly after treatment. All fecal samples were immediately stored at −20°C for later DNA extraction. Total genomic DNA was isolated from fecal samples as described [21] and kept for later time-course study. QIAamp DNA Stool Mini Kit (QIAGEN) was used to extract the fecal genomic DNA from experimental mice and kept for later pyrosequencing.

### Enterobacterial repetitive intergenic consensus (ERIC)-PCR and data analysis

ERIC sequences reside in the genome of various bacterial species in addition to enterobacteria [66]. As described in our previous study [21], ERIC-PCR was performed to profile the gut microbiota by using fecal genomic DNA from different treatment groups. Partial least squares discriminant analysis (PLS-DA) was applied to visualize the changes of microbial composition before and after treatments using SIMCA-P 12.0 tool (Umetrics, Umea, Sweden) for which the confidence level was set at 95% (P<0.05).

### 16S rRNA gene pyrosequencing of fecal DNA samples and data analysis

Five fecal samples randomly picked from each experimental group on week 8 were subjected to further analysis by using 16S rRNA gene pyrosequencing as our previous method with slight modification [21]. Briefly, 0.1 μg/μl BSA was added to enhance the PCR efficiency, and PCR was performed for each sample in a final reaction volume of 20 ul comprising 100 ng extracted DNA. Amplicon libraries were quantified, emulsion-PCR and pyrosequencing using titanium chemistry on the GS Junior System (454 Life Sciences Corp., Branford, CT, USA) was carried out as detailed by the manufacturer. Pyrosequencing data were processed and analyzed using the Quantitative Insights Into Microbial Ecology software (QIIME version 1.5.0) [67]. The raw 454 pyrosequencing data were deposited in NCBI's Sequence Read Archive (SRA) database under accession number of SRP057080. The differences in overall microbiota composition between compared samples were determined using the unweighted UniFrac metric. A matrix of pairwise distances between communities was constructed and used to generate Principal Coordinates Analysis (PCoA) plots. Linear discriminant analysis (LDA) effect size (LEfSe) method [68] was used to evaluate the key phylotypes responsible for the observed differences between microbial communities. The alpha value used for the algorithm of LEfSe was internally set at 0.05, which corresponded to 95% confidence level (P<0.05) to detect features with significant differential abundance and to test the biological consistency.

### Gut samples collection and polyp counting

At the end of the experiment, all mice were sacrificed and the intestinal tract was removed. Small intestine and colon were divided at cecal junction. 2 cm of small intestine and colon were cut from the adjacent cecum, rinsed with PBS and then fixed in 10% formalin for later histological sections. The remaining part of colon and 8 cm of distal small intestine were used for mucosal scrapings. Other part of the intestinal tract was opened longitudinally and rinsed with PBS and then fixed in 10% formalin. The number and sizes of polyps in the intestine were determined with a dissecting microscope after methyl blue staining.

### Quantitative reverse transcription polymerase chain reaction (qRT-PCR)

RNA was isolated from mucosal scrapings samples using TRIzol reagent (Invitrogen, Carlsbad, CA, USA) according to the manufacturer's instructions. First-strand cDNA was synthesized from 5 μg of total RNA using random primers and SuperScript II reverse transcriptase (Invitrogen, Carlsbad, CA, USA). qRT-PCR was performed to measure changes in mRNA expression using Applied Biosystems ViiA™ 7 PCR system (Carlsbad, CA, USA). The sequences of the primers used were listed in Supplementary Table 1. Briefly, the qRT-PCR was carried out using Power SYBR^®^ Green PCR Maser Mix (Applied Biosystems Inc., Carlsbad, CA, USA). The amplification conditions were as follow: 95°C for 10 min, followed by 40 cycles of 95°C for 15 s and 60°C for 1 min. Six samples were used for each experimental group. Hypoxanthine-guanine phosphoribosyl transferase 1 (Hprt1) was used as an internal control. Before we applied Hprt1 to qRT-PCR data analysis, we compared the expression stability of Hprt1 and β-actin, and the two reference genes showed a similar expression pattern among different samples. On the other hand, the expression of *dsrA* gene was also carried out by qRT-PCR using 5 ng fecal genomic DNA, and normalized to that of the total fecal bacteria, which was detected by 16S rRNA gene. The 2^−ΔΔCt^ method was applied to calculate the fold change of relative gene expression. ΔΔCt = (Ct_treatment_target gene_ - Ct_treatment_reference gene_) - (Ct_control_target gene_ - Ct_control_reference gene_).

### Mucosal protein extraction

The protein of mucosal scraping samples from small intestine of colon were extracted by homogenization, and followed by sonication in Raybiotech cell lysis buffer with protease inhibitors. Protein concentration was determined by DC Protein Assay (Bio-Rad, Hercules, CA).

### Cytokine array

Mucosal lysates from the same experimental group were pooled together and applied to a mouse cytokine array (RayBiotech, Inc.). Each cytokine was represented in duplicate on the membrane. Two independent experiments were performed to evaluate the expression level of various cytokines. The intensity of signal was quantified by densitometry (ImageJ, NIH). The positive control was used to normalize the results from different membranes being compared.

### Western blot

Western blot analysis was performed using standard methods on the mucosal protein lysates from individual experimental mice. Immunodetection was performed using specific antibodies against p-Stat3 (1:1000, cell signaling #9138), Stat3 (1:1000, cell signaling #9132), p-Src (1:1000, cell signaling #6943) and Src (1:1000, cell signaling #2123) purchased from Cell Signaling Technology (Beverly, MA), and GAPDH (1:1000, sc-20357) purchased from Santa Cruz Biotechnology (Santa Cruz, CA).

### Histology and immunohistochemistry

5 μm thick paraffin sections were used for hematoxylin and eosin (H&E) staining, Alcian blue-staining, and immunohistochemical staining using standard procedures. Immunohistochemistry was performed using antibodies against Lysozyme (1:200, A0099, DAKO), E-Cadherin (1:200, #3195S, Cell Signaling), N-Cadherin (1:100, 610920, BD), Stat3 (1:200, #9139, Cell Signaling), beta-catenin (1:50, sc-7963, Santa Cruz), IL-4 (1:100, PAB16160, Abnova), iNOS (1:200, ab129372, Abcam), Arginase I (1:100, 610708, BD), and LSAB+System-HRP kit (K0679, DAKO). The slides were mounted and viewed on a Nikon Eclipse 80i microscope. Images were photographed with a SPOT RT3 CCD camera and SPOT Advanced software (Diagnostic Instruments, Sterling Heights, MI, USA).

### Statistical analysis

The data is presented as mean ± SEM, and statistical comparisons were performed using one-way ANOVA followed by Dunnett's post test with the GraphPad Prism version 5.00 (GraphPad Software, San Diego, CA, USA) or Student's t-test at *P* < 0.001(***), *P* < 0.01(**) or *P* < 0.05(*).

## SUPPLEMENTARY DATA FIGURES AND TABLE



## Abbreviations

*APC*: adenomatous polyposis coli; BLAST: basic local alignment search tool;
CMC: carboxymethyl cellulose;
CRC: colorectal cancer;
*dsrA*: dissimilatory (bi)sulfite reductase;
ERIC: enterobacterial repetitive intergenic consensus;
Gp: *Gynostemma pentaphyllum*;
GpS: *Gynostemma pentaphyllum* saponins;
H_2_S: hydrogen sulfide;
H&E: hematoxylin and eosin;
Hprt1: hypoxanthine-guanine phosphoribosyl transferase 1;
IgA: immunoglobulin A;
IHC: immunohistochemistry;
ISC: intestinal stem cell;
LDA: linear discriminant analysis;
LEfSe: linear discriminant analysis effect size;
MCP: monocyte chemoattractant protein;
PCoA: principal coordinates analysis;
pIgR: polymeric immunoglobulin receptor;
PLS-DA: partial least squares discriminant analysis;
QIIME: quantitative insights into microbial ecology;
qRT-PCR: quantitative reverse transcription polymerase chain reaction;
SRA: sequence read archive;
SRB: sulfate-reducing bacteria;
STAT3: signal transducer and activator of transcription 3;
sTNFRI: soluble tumor necrosis factor receptor I;
TAM: tumor associated macrophage;
WT: wild-type
