# Supplementary material for: Triterpenoid herbal saponins enhance beneficial bacteria, decrease sulfate-reducing bacteria, modulate inflammatory intestinal microenvironment and exert cancer preventive effects in ApcMin/+ mice
Source: Oncotarget. 2016 Apr 21;7(21):31226–42. doi: 10.18632/oncotarget.8886 (PMC5058752; doi:10.18632/oncotarget.8886)
Supplement: Supplementary file 1 [file oncotarget-07-31226-s001.pdf]

SUPPLEMENTARY DATA

REFERENCES

1. Liu X, Lu R, Wu S, Sun J: Salmonella regulation of intestinal stem cells through the Wnt/beta-catenin pathway. FEBS Lett 2010; 584:911-916.
2. Willems E, Mateizel I, Kemp C, Cauffman G, Sermon K, Leyns L: Selection of reference genes in mouse embryos and in differentiating human and mouse ES cells. Int J Dev Biol 2006; 50:627-635.
3. Dostal A, Baumgartner J, Riesen N, Chassard C, Smuts CM, Zimmermann MB, Lacroix C: Effects of iron supplementation on dominant bacterial groups in the gut, faecal SCFA and gut inflammation: a randomised, placebo-controlled intervention trial in South African children. Br J Nutr 2014; 112:547-556.
4. Shanahan MT, Carroll IM, Grossniklaus E, White A, von Furstenberg RJ, Barner R, Fodor AA, Henning SJ, Sartor RB, Gulati AS: Mouse Paneth cell antimicrobial function is independent of Nod2. Gut 2014; 63:903-910.
5. Salzman NH, Hung K, Haribhai D, Chu H, Karlsson-Sjoberg J, Amir E, Teggatz P, Barman M, Hayward M, Eastwood D et al: Enteric defensins are essential regulators of intestinal microbial ecology. Nat Immunol 2010; 11:76-83.
6. Ren W, Chen S, Yin J, Duan J, Li T, Liu G, Feng Z, Tan B, Yin Y, Wu G. Dietary arginine supplementation of mice alters the microbial population and activates intestinal innate immunity. J Nutr 2014; 144:988-995.
7. Nakanishi Y, Nakatsuji M, Seno H, Ishizu S, Akitake-Kawano R, Kanda K, Ueo T, Komekado H, Kawada M, Minami M et al: COX-2 inhibition alters the phenotype of tumor-associated macrophages from M2 to M1 in ApcMin/+ mouse polyps. Carcinogenesis 2011; 32:1333-1339.
8. Li Y, Kundu P, Seow SW, de Matos CT, Aronsson L, Chin KC, Karre K, Pettersson S, Greicius G: Gut microbiota accelerate tumor growth via c-jun and STAT3 phosphorylation in APCMin/+ mice. Carcinogenesis 2012; 33:1231-1238.
9. Ma F, Li Y, Jia L, Han Y, Cheng J, Li H, Qi Y, Du J: Macrophage-stimulated cardiac fibroblast production of IL-6 is essential for TGF beta/Smad activation and cardiac fibrosis induced by angiotensin II. PLoS One 2012; 7:e35144.
10. Lam W, Bussom S, Guan F, Jiang Z, Zhang W, Gullen EA, Liu SH, Cheng YCL: The four-herb Chinese medicine PHY906 reduces chemotherapy-induced gastrointestinal toxicity. Sci Transl Med 2010; 2:45ra59.
11. Peng DJ, Zeng M, Muromoto R, Matsuda T, Shimoda K, Subramaniam M, Spelsberg TC, Wei WZ, Venuprasad K: Noncanonical K27-linked polyubiquitination of TIEG1 regulates Foxp3 expression and tumor growth. J Immunol 2011; 186:5638-5647.

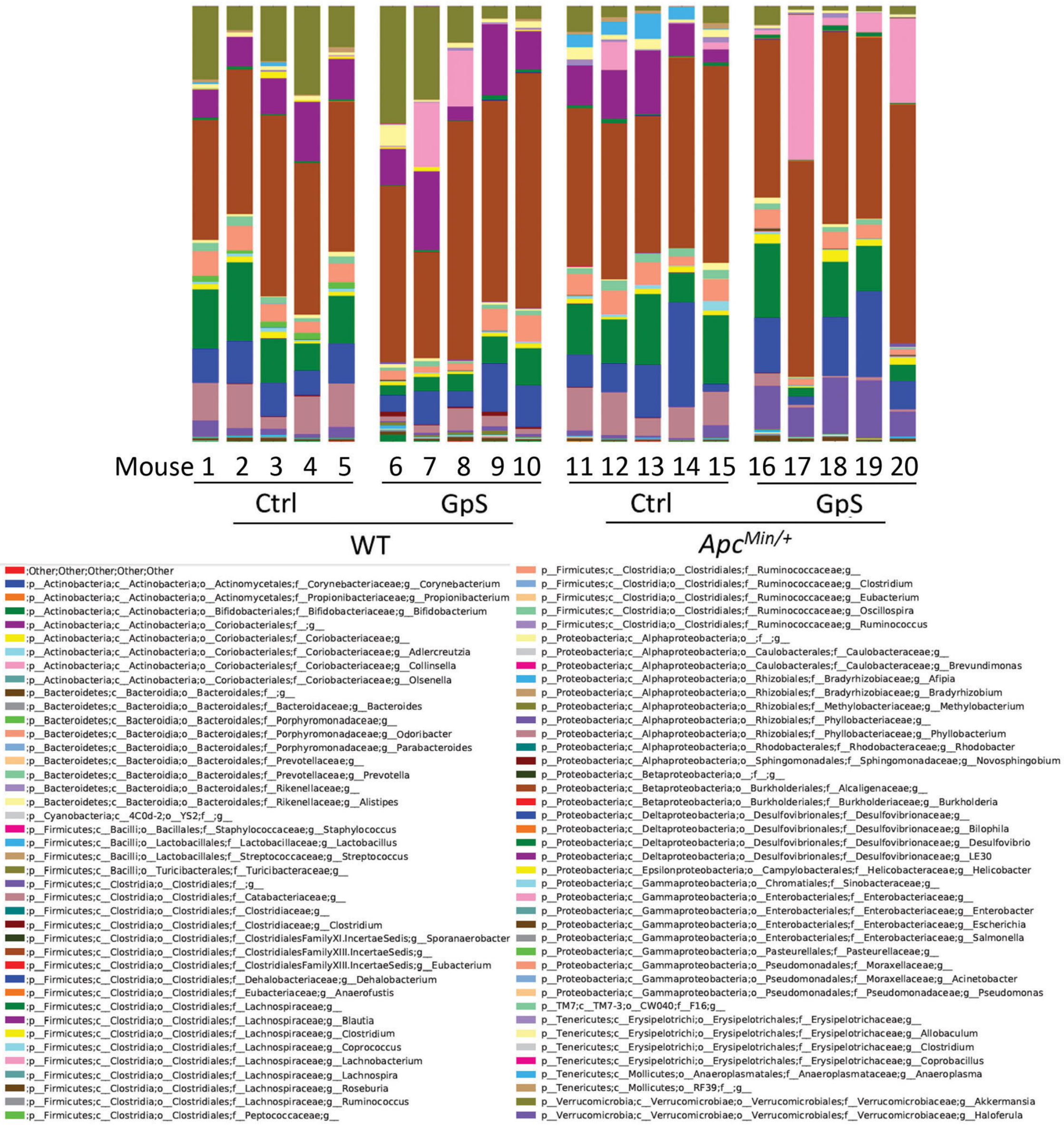

Supplementary Figure S1: A bar chart of the relative abundance of bacterial genera in the individual mice of different treatment groups.

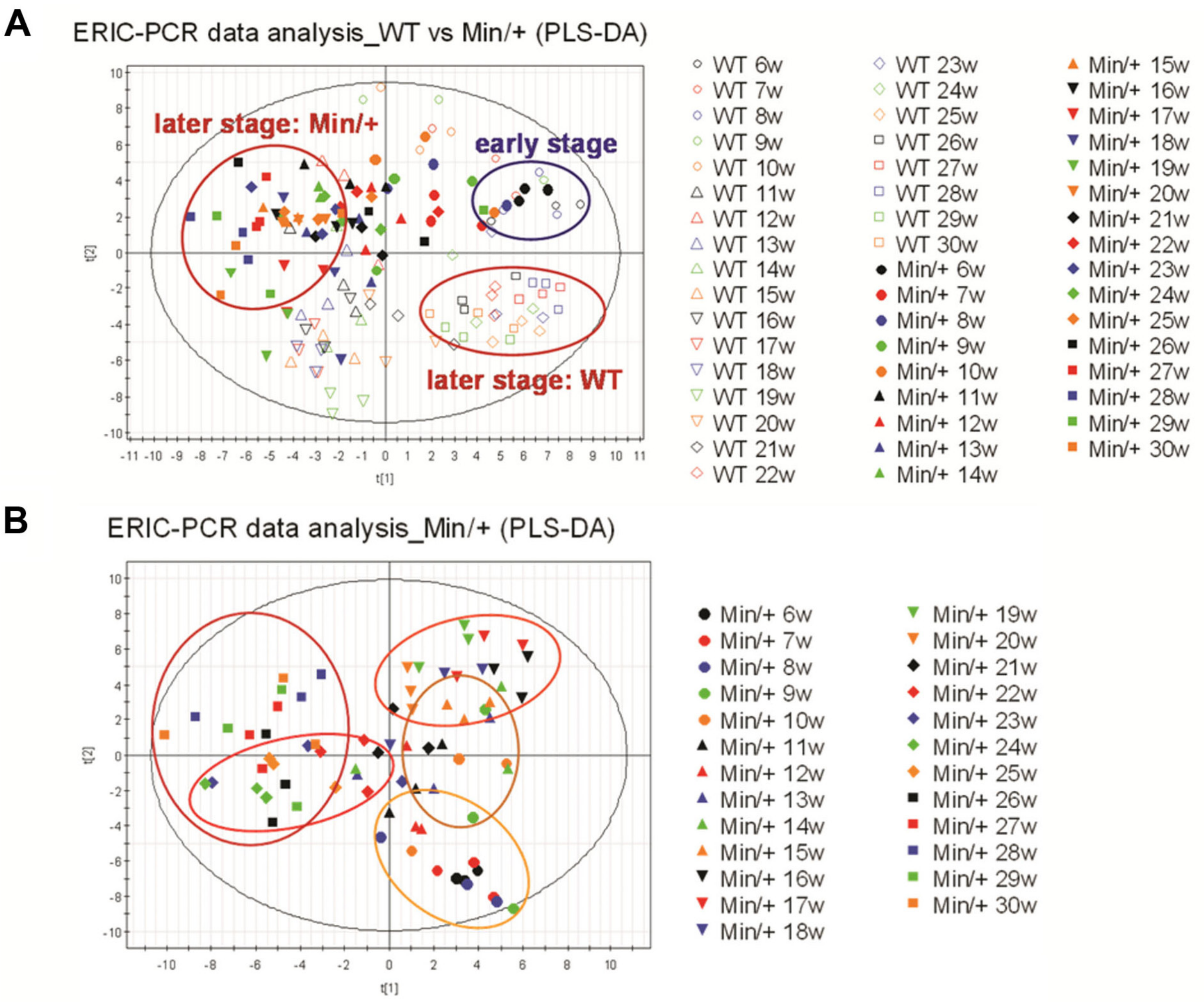

Supplementary Figure S2: The time course PLS-DA plots of ERIC-PCR DNA profile. A. *Apc*$^{Min/+}$ mice versus WT. Open symbols: WT; Solid symbols: *Apc*$^{Min/+}$ mice. **B.** *Apc*$^{Min/+}$ mice at different disease stages. Different symbol shapes represented for different disease stages. The microbial profiles of *Apc*$^{Min/+}$ mice and their WT littermates were characterized from 6 weeks to 30 weeks of age for 25 consecutive weeks. The fecal samples were prepared for genomic DNA and subjected for ERIC-PCR. Gel pictures were digitized by Image Lab 3.0 system (Bio-Rad). Based on the distance and the intensity of each DNA band, SIMCA-P 12.0 tool was applied to obtain the PLS-DA score plots. WT: n=3; *Apc*$^{Min/+}$ mice: n=3.

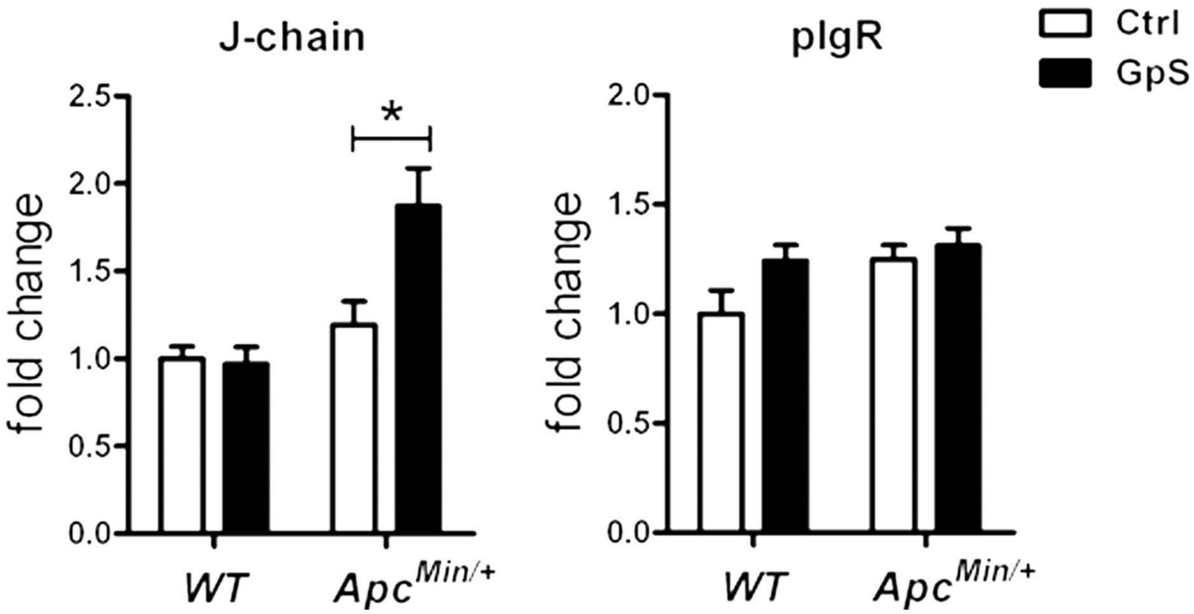

Supplementary Figure S3: The relative mRNA expression of J-chain and pIgR. J-chain is known to be critical for polymeric immunoglobulin receptor (pIgR)-mediated epithelial transport of IgA. Data were normalized to the expression of reference gene, and expressed as fold change of the WT control group. Data is presented as the mean ± SEM (* $P < 0.05$, GpS versus control samples); n=6/group.

Supplementary Table S1: Primers for Quantitative Real-Time PCR

Target gene	Nucleotide sequence of primers (5' to 3')		References
	Forward	Reverse	
β-actin	TGTTACCAACTGGGACGACA	CTGGGTCATCTTTTCACGGT	[1]
Hprt1	TCAGTCAACGGGGGACATAAA	GGGGCTGTACTGCTTAACCAG	[2]
dsrA	CTGCGAATATGCCTGCTACA	TGGTCGARCTTGATGTCGTC	[3]
PanCrp	GGTGATCATCAGACCCCAGCATCAGT	AAGAGACTAAAACTGAGGAGCAGC	[4]
Lyz 1 (P-lysozyme)	GCCAAGGTCTACAATCGTTGTGAGTTG	CAGTCAGCCAGCTTGACACCACG	[5]
Lyz 2 (M-lysozyme)	GGCTGGCTACTATGGAGTCAGCCTG	GCATTCACAGCTCTTGGGGTTTTG	[5]
MUC2	CCCAGAAGGGACTGTGTATG	TTGTGTTCGCTCTTGGTCAG	[6]
MUC4	GTCTCCCATCACGGTTCAGT	TGTCATTCCACACTCCCAGA	[6]
J-chain	GAACTTTGTATACCATTTGTCAGACG	CTGGGTGGCAGTAACAACCT	[6]
pIgR	AGTAACCGAGGCCTGTCCTT	GTCACTCGGCAACTCAGGA	[6]
iNOS	GTTCTCAGCCCAACAATACAAGA	GTGGACGGGTCGATGTCAC	[7]
CXCL10	CCAAGTGCTGCCGTCATTTTC	GGCTCGCAGGGATGATTTCAA	[7]
Arginase 1	TGGCTTGCGAGACGTAGAC	GCTCAGGTGAATCGGCCTTTT	[7]
Ym1	TTATCCTGAGTGACCCTTCTAAG	TCATTACCCTGATAGGCATAGG	[7]
Trem2	CTGGAACCGTCACCATCACTC	CGAAACTCGATGACTCCTCGG	[7]
MR	GCTGAATCCCAGAAATTCCGC	ATCACAGGCATACAGGGTGAC	[7]
IFN-γ	ATGAACGCTACACACTGCATC	CCATCCTTTTGCCAGTTCCTC	[7]
TNF-α	CAAATGGCCTCCCTCTCAT	CTCCTCCACTTGGTGGTTTG	[8]
IL-12	ACTCTGCGCCAGAAACCTC	CACCCTGTTGATGGTCACGAC	[7]
IL-4	GGTCTCAACCCCCAGCTAGT	GCCGATGATCTCTCTCAAGTGAT	[7]
IL-10	GCTCTTACTGACTGGCATGAG	CGCAGCTCTAGGAGCATGTG	[7]
IL-13	GGATATTGCATGGCCTCTGTAAC	AACAGTTGCTTTGTGTAGCTGA	[7]
IL-1β	GCTGAAAGCTCTCCACCTCA	GGCCACAGGTATTTTGTCGT	[8]
IL-6	CTTCCATCCAGTTGCCTTCTTG	AATTAAGCCTCCGACTTGTGAAG	[9]
IL-18	CCTGACATCTTCTGCAACCT	TTCCGTATTACTGCGGTTGT	[10]
IL-23	GCACCTGCTTGACTCTGACA	ATCCTCTGGCTGGAGGAGTT	[8]
Foxp3	CCCATCCCCAGGAGTCTTG	ACCATGACTAGGGGCACTGTA	[11]
IL-17	TTTAACTCCCTTGGCGCAAAA	CTTTCCCTCCGCATTGACAC	[11]
